# Clinical characteristics, co-detection patterns and prognostic risk factors of human metapneumovirus infection in children: a retrospective study

**DOI:** 10.3389/fcimb.2026.1868123

**Published:** 2026-07-07

**Authors:** Wen Li, Chi Zhu, Mengfei Yu, Qi Jia, Xin Dong, Yang Wang, Zhenghong Qi

**Affiliations:** 1 Department of Pediatric Infectious Diseases, Hangzhou Children’s Hospital, Hangzhou, China; 2 Department of Clinical Laboratory, Hangzhou Adicon Medical Laboratory Co. Ltd., Hangzhou, China

**Keywords:** coinfection pattern, human metapneumovirus, inflammatory index, pediatric infection, prognostic risk factor

## Abstract

**Objective:**

To investigate clinical characteristics, co-detection patterns, and prognostic risk factors of human metapneumovirus (hMPV) infection in children.

**Methods:**

Retrospective study of 621 children with laboratory-detected hMPV infection. Clinical data, laboratory indicators, and co-detection status were analyzed. Multivariate logistic regression identified independent risk factors for severe pneumonia.

**Results:**

Median age was 3.50 years (IQR 1.75–4.75). Of 621 patients, 399 (64.3%) had Single-detection and 222 (35.7%) co-detection, with viral-viral co-detection dominant (68.9%) and rhinovirus the most common co-pathogen (39.6%). Only platelet count and aspartate aminotransferase differed significantly between groups (both P<0.01), with all inflammatory markers improving post-treatment (all P<0.001). Viral-bacterial co-detection was associated with longer hospitalization (median 6.5 days) and higher severe pneumonia rate (14.5%, both P<0.01). Multivariate analysis identified *Streptococcus pneumoniae* co-detection (aOR=8.43, 95%CI:1.50–47.32), prolonged fever duration (aOR=1.19, 95%CI:1.02–1.38), and procalcitonin ≥0.5 ng/mL (aOR=4.27, 95%CI:1.76–10.33) as independent risk factors for severe pneumonia. Older age (3–5 years: aOR=0.20; >5 years: aOR=0.03) showed significant protective effects.

**Conclusion:**

hMPV infection is most prevalent in preschool children. Viral-bacterial co-detection was associated with worse clinical outcomes. *S. pneumoniae* co-detection, prolonged fever, and elevated procalcitonin were independently associated with increased risk, while older age was associated with reduced risk. These findings support early risk stratification and targeted intervention.

## 1 Introduction

Human metapneumovirus (hMPV), a member of the *Pneumoviridae* family, is an important pathogen causing acute lower respiratory tract infection (LRTI) in children, especially in infants and preschool children (Khales et al., 2025). First identified in the Netherlands in 2001, hMPV has been found to circulate worldwide, with a prevalence rate of 5%-15% in pediatric respiratory infection cases, and its epidemic characteristics are similar to those of respiratory syncytial virus (RSV, Van den Hoogen et al., 2001). hMPV infection can cause a variety of clinical manifestations, from mild upper respiratory tract symptoms to severe pneumonia, respiratory failure and even death, and is one of the main causes of pediatric hospitalization due to respiratory infection (Ribó-Molina et al., 2024).

In clinical practice, hMPV infection is often accompanied by co-detection with other respiratory pathogens, including viruses, bacteria and atypical pathogens, which may affect the clinical severity and prognosis of the disease (Howard et al., 2021; Bhattacharya et al., 2025; Gnanasekaran et al., 2025). Previous studies have shown that the co-detection rate of hMPV in children is about 6%-23%, and viral-viral co-detection is the main type, but the clinical impact of different co-detection patterns on hMPV infection remains controversial (Evelyn et al., 2019; Aradhana et al., 2025; Amarin et al., 2026). In addition, inflammatory markers are important indicators for evaluating the severity of respiratory infections and guiding clinical treatment. Conventional inflammatory markers such as C-reactive protein (CRP) and procalcitonin (PCT) have been widely used in clinical practice, while novel inflammatory indices such as neutrophil-to-lymphocyte ratio (NLR), platelet-to-lymphocyte ratio (PLR) and immune balance index (IBI) have shown good application value in the prognostic assessment of various infectious diseases in recent years (Üçsular et al., 2020; Li et al., 2021; Pantea et al., 2024). However, the value of these novel inflammatory indices in the prognostic assessment of hMPV-infected children has not been fully clarified.

At present, most studies on hMPV infection in children focus on clinical characteristics and epidemic trends, and there are few large-sample studies systematically analyzing the co-detection patterns, dynamic changes of inflammatory markers and comprehensive prognostic risk factors. Therefore, this study retrospectively analyzed the clinical data of 621 children with laboratory-detected hMPV infection, aiming to explore the clinical characteristics, co-detection patterns and laboratory features of hMPV infection in children, and identify the independent risk factors for severe pneumonia, so as to provide evidence-based reference for clinical risk stratification, targeted etiological screening, and personalized intervention of hMPV infection in children.

## 2 Methods

### 2.1 Study design and setting

This retrospective cohort study was conducted at Hangzhou Children’s Hospital, a tertiary pediatric hospital in Hangzhou, between July 2023 and June 2025. The study protocol was approved by the Institutional Review Board (No.2021-059). In accordance with ethical guidelines for retrospective studies, informed consent was waived owing to the anonymization of all participant data. Data anonymization was performed by assigning unique study identifiers to replace personal information. All direct identifiers (name, medical record number, contact information) were removed and replaced with coded identifiers. Date of birth was converted to age in months/years. Admission and discharge dates were converted to relative time variables. Data anonymization followed the Health Insurance Portability and Accountability Act (HIPAA) Safe Harbor method.

Consecutive patients aged <14 years with laboratory-detected hMPV infection were eligible for inclusion. hMPV infection was diagnosed by detection of hMPV nucleic acid in respiratory specimens (sputum, nasopharyngeal swab or bronchoalveolar lavage fluid [BALF]) using multiplex polymerase chain reaction (PCR) or targeted next-generation sequencing (tNGS) assays. Patients were excluded if they had incomplete medical records, were transferred from other hospitals >48 hours after admission, or had specimens collected >7 days after symptom onset.

### 2.2 Data collection and clinical definitions

Demographic characteristics, medical history, and clinical manifestations were retrospectively retrieved from standardized electronic medical records. Clinical data included age, sex, underlying medical conditions, duration of symptoms prior to admission, vital signs, physical examination findings, and laboratory parameters.

Disease severity was classified as follows: Non-LRTI: Upper respiratory tract infection without radiographic evidence of lower tract involvement; Bronchopneumonia: Clinical symptoms plus radiographic evidence of patchy or diffuse infiltrates; Pneumonia: Lobar or segmental consolidation on chest radiography; Severe pneumonia: Presence of any of the following (Khales et al., 2025): respiratory rate >70 breaths/minute (infants) or >50 breaths/minute (older children, Van den Hoogen et al., 2001); oxygen saturation <92% on room air (Ribó-Molina et al., 2024); requirement for supplemental oxygen (Gnanasekaran et al., 2025); mechanical ventilation; or (Bhattacharya et al., 2025) admission to pediatric intensive care unit (PICU, WHO, 2013; The Subspecialty Group of Respiratory, the Society of Pediatrics, Chinese Medical Association, 2024).

hMPV single detection: Only hMPV nucleic acid was positive in respiratory specimens, with no other pathogens detected. hMPV co-detection: hMPV nucleic acid was positive combined with one or more other respiratory pathogens detected; further stratified into viral-viral co-detection (hMPV + other viruses) and viral-atypical bacterial/bacterial co-detection (hMPV + *Mycoplasma pneumoniae* (M.P), *Streptococcus pneumoniae* (S.P), *H. influenzae*, or *C. pneumoniae*). Elevated inflammatory markers: CRP ≥20 mg/L, PCT ≥0.5 ng/mL. Age divided into four groups according to age at admission: ≤1 year, 1–3 years, 3–5 years, and >5 years. Treatment protocols: All patients received supportive care including oxygen therapy when indicated (SpO2 <92% on room air). Antiviral therapy was not routinely administered for hMPV. Antibiotic use was at the discretion of attending physicians based on clinical judgment: 312 patients (50.2%) received empirical antibiotics, with 89.4% receiving β-lactams and 45.2% receiving macrolides. Corticosteroids were used in 28 patients (4.5%) for severe bronchospasm. Post-treatment biomarkers were measured at 48–72 hours after initiation of treatment. Data on prior antibiotic use (within 7 days before admission) and vaccination status were collected when available from medical records; however, these data were incomplete.

Novel inflammatory indices calculation: NLR = neutrophil count/lymphocyte count; PLR = PLT count/lymphocyte count; IBI = (neutrophil count × monocyte count)/(lymphocyte count × PLT count, Kikuchi et al., 2025); PWR (Platelet-to-White blood cell Ratio) = PLT count/WBC count.

### 2.3 Specimen collection and processing

Respiratory specimens were collected within 24 hours of hospital admission, prior to initiation of empirical antimicrobial therapy. Specimen types included: (i) oropharyngeal swabs collected using sterile flocked nylon swabs from the posterior pharynx and tonsillar pillars; (ii) Sputum samples: expectoration or suctioning from lower respiratory tract in children >3 years; and (iii) BALF: obtained during flexible bronchoscopy in patients with severe or complicated pneumonia. All specimens were immediately transported to the laboratory within 2 hours. For quality control, specimens with insufficient volume (<1 mL), visible blood contamination, or processing delays >4 hours were excluded from molecular analysis. All retained samples were subjected to routine culture, multiplex PCR, or tNGS testing.

### 2.4 Targeted next-generation sequencing and multiplex PCR

Upon receipt, all respiratory specimens were checked for sample identification, type, collection time, and storage conditions. Nucleic acid extraction was performed using a magnetic bead-based method to obtain total nucleic acid from 200 μL of each specimen. Nucleic acid concentration and purity were assessed by spectrophotometry (A260/A280 ratio 1.8–2.0). Targeted amplification was performed using pathogen-specific primers designed against conserved gene regions of respiratory pathogens including hMPV, respiratory syncytial virus (RSV), influenza viruses (A and B), parainfluenza viruses (1–4), rhinoviruses (A, B, C), adenoviruses, human bocavirus, coronaviruses (NL63, OC43, HKU1, 229E), enteroviruses, and herpesviruses. Bacterial targets included S.P, *H. influenzae*, *S. aureus*, *M. catarrhalis*, *Klebsiella species*, *Pseudomonas species*, and atypical pathogens (M.P, *C. pneumoniae*). Sequencing libraries were constructed following PCR amplification and sequenced on theSalus Pro with a mean sequencing depth ≥500×. Raw sequencing data underwent quality control (Q30 ≥85%), adapter trimming, and alignment against a pathogen reference database. Pathogen detection criteria were predefined as (1): unique mapped reads ≥100 for viruses (2); unique mapped reads ≥500 for bacteria (3); coverage depth ≥10×; and (4) detection in both duplicate PCR reactions. These thresholds were established to balance analytical sensitivity with specificity, minimizing false-positive detection of background contaminants or colonizing organisms. The viral threshold (≥100 reads) is substantially higher than the minimum criteria (normalized read count ≥3 or ≥10) reported in recent pediatric tNGS studies (Zhang et al., 2018; Li et al., 2025), ensuring robust detection of true viral pathogens while filtering out low-level environmental or reagent-derived sequences. The bacterial threshold (≥500 reads) accounts for the higher background abundance of bacterial DNA in respiratory specimens and aligns with the principle that clinically significant bacterial infections typically present with higher microbial loads than viral infections (Yang et al., 2025; Chen et al., 2026). The coverage depth requirement (≥10×) ensures reliable genomic representation for accurate species identification, consistent with established tNGS quality standards (Gu et al., 2025). The duplicate PCR requirement further reduces stochastic amplification artifacts. Final pathogen identification was determined by integrating sequencing data with clinical information, with contaminant sequences excluded. Common laboratory contaminants and oral commensal organisms were excluded unless they met additional relative abundance criteria (>80% of genus-level reads or sequence coverage 10-fold higher than other microorganisms (Ding et al., 2025).

The detection range of multiplex PCR (HEALTH BioMed, China) included rhinovirus, human bocavirus, parainfluenza virus, M.P, coronavirus (OC43, HKU1, 229E, NL63), respiratory syncytial virus, influenza A virus, hMPV, adenovirus, influenza B virus, *C. pneumoniae*. Laboratory personnel performing molecular testing were blinded to clinical outcomes and co-detection status. Technicians processed samples in batches with automated nucleic acid extraction to minimize handling bias.

### 2.5 Statistical analysis

Statistical analysis was performed using SPSS 26.0 software (IBM Corp., Armonk, NY, USA) and GraphPad Prism 9.0 (GraphPad Software, Inc., La Jolla, CA, USA). Normality of continuous variables was tested by the Shapiro-Wilk test. Non-normally distributed continuous variables were expressed as median (interquartile range [IQR]), and inter-group comparisons were performed using the Mann-Whitney U test (two groups) or Kruskal-Wallis H test (three or more groups). Categorical variables were expressed as number (percentage, n (%)), and inter-group comparisons were performed using the chi-square (χ²) test; Fisher’s exact test was used when the expected frequency was <5.

Multivariate logistic regression analysis was used to identify independent risk factors for severe pneumonia: severe pneumonia (1 = yes, 0 = no) was set as the dependent variable; variables with P<0.1 in univariate analysis (Infection Type, Mixed infection, S.P co-detection, CRP, PCT) were included as independent variables; potential confounders (age, gender, fever duration) were adjusted in the model. Results were expressed as adjusted odds ratio (aOR) and 95% confidence interval (CI). Multicollinearity was assessed using Variance Inflation Factor (VIF); variables with VIF >5.0 were considered to have multicollinearity issues. Model goodness-of-fit was assessed using the Hosmer-Lemeshow test, and discriminatory ability was evaluated using the C-statistic (area under the ROC curve). For continuous variables, linearity of the logit was assessed using restricted cubic splines with 3 knots. Non-significant non-linearity tests supported linear modeling. For variables with <5% missing data (n=12 patients for complete blood count parameters), complete case analysis was used. Sensitivity analysis using multiple imputation (5 imputations) for missing laboratory values showed consistent results with the complete case analysis. Given the exploratory nature of secondary analyses (inflammatory markers, co-detection patterns), we did not apply Bonferroni correction for multiple comparisons to avoid excessive Type II error. However, we acknowledge that some associations may be chance findings and recommend cautious interpretation. For the primary multivariate analysis testing the pre-specified hypothesis about severe pneumonia risk factors, no multiple comparison correction was needed.

Graphs were plotted with GraphPad Prism 9.0, including bar charts for inflammatory marker changes, co-detection spectrum distribution, age-related co-detection trends, and clinical outcomes of different co-detection patterns; a forest plot was used to display the results of multivariate logistic regression. A two-sided *P* < 0.05 was considered statistically significant.

## 3 Results

### 3.1 Patient characteristics and hMPV infection patterns

A total of 621 children with laboratory-detected hMPV infection were enrolled in this study (**Table 1**). The median age was 3.50 years (IQR 1.75–4.75), and 337 (54.3%) patients were male. The age distribution was as follows: ≤1 year (93 cases, 15.0%), 1–3 years (179 cases, 28.8%), 3–5 years (215 cases, 34.6%), and >5 years (134 cases, 21.6%). Regarding clinical diagnosis, the majority of patients (517 cases, 83.3%) were diagnosed with bronchopneumonia, 49 cases (7.9%) with pneumonia, 38 cases (6.1%) with severe pneumonia, and 17 cases (2.7%) with non-lower respiratory tract infection (non-LRTI, mainly upper respiratory tract infection). Respiratory specimens for hMPV detection were predominantly oropharyngeal swabs (589 cases, 95.0%), with sputum (24 cases, 3.9%) and BALF (7 cases, 1.1%) as minor specimen types. The most common clinical symptoms were cough (602 cases, 97.4%), rhinorrhea (202 cases, 32.7%), wheezing (132 cases, 21.4%), and dyspnea (41 cases, 6.6%); all symptoms showed no significant differences between groups (all P>0.05).

**Table 1:** Baseline characteristics and clinical outcomes of 621 pediatric patients with hMPV infection.

Variables	All patients (n = 621)	Single-detection (n = 399)	Co-detection (n = 222)	Statistic	P
Baseline characteristics					
Age, years, M (Q^1^, Q^3^)	3.50 (1.75, 4.75)	3.58 (1.83, 4.75)	3.21 (1.60, 4.96)	Z=-0.40	0.690
Age group, n (%)				χ²=5.14	0.162
≤1	93 (14.98)	55 (13.78)	38 (17.12)		
1-3	179 (28.82)	114 (28.57)	65 (29.28)		
3-5	215 (34.62)	150 (37.59)	65 (29.28)		
>5	134 (21.58)	80 (20.05)	54 (24.32)		
Boy, n(%)	337 (54.27)	219 (54.89)	118 (53.15)	χ²=0.17	0.678
Diagnosis, n(%)				χ²=5.90	0.116
Non-LRTI	17 (2.74)	7 (1.75)	10 (4.50)		
Bronchopneumonia	517 (83.25)	341 (85.46)	176 (79.28)		
Pneumonia	49 (7.89)	28 (7.02)	21 (9.46)		
Severe pneumonia	38 (6.12)	23 (5.76)	15 (6.76)		
Sample type, n(%)				-	0.225
Throat swab	589 (95.00)	383 (95.99)	206 (93.21)		
Sputum	24 (3.87)	13 (3.26)	11 (4.98)		
BALF	7 (1.13)	3 (0.75)	4 (1.81)		
Symptoms					
Cough, n(%)	602 (97.41)	386 (97.23)	216 (97.74)	χ²=0.15	0.703
Wheezing, n(%)	132 (21.36)	78 (19.65)	54 (24.43)	χ²=1.94	0.164
Dyspnea, n(%)	41 (6.63)	24 (6.05)	17 (7.69)	χ²=0.62	0.430
Rhinorrhea, n(%)	202 (32.74)	123 (31.06)	79 (35.75)	χ²=1.41	0.234
Vomiting, n(%)	60 (9.71)	44 (11.08)	16 (7.24)	χ²=2.39	0.122
Outcome					
Hospitalization duration, days, M (Q^1^, Q^3^)	4.00 (3.00, 6.00)	4.00 (3.00, 5.00)	5.00 (4.00, 6.75)	Z=-4.10	<0.001
Fever duration Before admission, days, M (Q^1^, Q^3^)	2.00 (0.50, 3.00)	2.00 (1.00, 4.00)	1.00 (0.00, 3.00)	Z=-2.54	0.011
Fever duration after admission, days, M (Q^1^, Q^3^)	1.00 (0.00, 2.00)	1.00 (0.00, 2.00)	1.00 (0.00, 2.00)	Z=-0.81	0.419
Fever duration, days, M (Q^1^, Q^3^)	4.00 (1.88, 5.00)	4.00 (2.00, 5.00)	3.00 (1.00, 5.00)	Z=-1.83	0.067
Oxygen supplementation, n(%)	31 (5.01)	20 (5.01)	11 (5.00)	χ²=0.00	0.995
PICU admission, n(%)	10 (1.61)	5 (1.25)	5 (2.26)	χ²=0.39	0.533

Z, Mann-Whitney test; χ², Chi-square test; Fisher exact.

LRTI, lower respiratory tract infection; BALF, bronchoalveolar lavage fluid; PICU, pediatric intensive care unit.

Of the 621 patients, 399 (64.3%) had hMPV single-detection and 222 (35.7%) had co-detectable pathogens (co-detection group). No significant differences were observed between the two groups in age (Z=-0.40, P = 0.690) or boy ratio (χ²=0.17, P = 0.678). For clinical outcomes, hospitalization duration was significantly longer in the co-detection group (5 days [4.00, 6.75]) compared with the single-detection group (4 days [3.00–5.00], Z=-4.10, P<0.001). Pre-admission fever duration was shorter in the co-detection group (1.0 [0.00–3.00]) than in the single-detection group (2.0 [1.00–4.00], Z=-2.54, P = 0.011), whereas total fever duration showed a trend toward shortening in the co-detection group without reaching statistical significance (Z=-1.83, P = 0.067). No between-group differences were found in oxygen supplementation rate (5.0% vs. 5.0%, χ²=0.00, P = 0.995) or PICU admission rate (2.3% vs. 1.3%, χ²=0.39, P = 0.533). Complications occurred in 12 patients (1.9%): pleural effusion (n=5), respiratory failure (n=4), and empyema (n=3). All complications occurred in the viral-bacterial co-detection group. Mechanical ventilation was required in 3 patients (0.48%), all in the viral-bacterial co-detection group. No deaths occurred during the study period.

### 3.2 Laboratory indicators

The comparison of baseline hematological, inflammatory, and organ function parameters between the single- and co-detection groups is presented in **Table 2**. For hematological indices, PLT was significantly higher in the co-detection group (253.5 [208.25–323.50]) than in the single-detection group (233.0 [196.00–283.75], Z=-3.12, P = 0.002), while white blood cell count (WBC), neutrophil count, lymphocyte count, and monocyte count showed no significant between-group differences (all P>0.05).

**Table 2:** Comparison of laboratory parameters between single-detection and co-detection groups in children with hMPV infection.

Variables	All patients (n = 621)	Single-detection (n = 399)	Co-detection (n = 222)	Statistic	P
WBC, ×10^9^/L, M (Q^1^, Q^3^)	6.96 (5.17, 8.91)	6.73 (5.16, 8.74)	7.35 (5.25, 9.58)	Z=-1.95	0.051
Neutrophil count, ×10^9^/L, M (Q^1^, Q^3^)	3.22 (2.05, 4.94)	3.13 (2.01, 4.71)	3.46 (2.12, 5.42)	Z=-1.54	0.124
PLT, ×10^9^/L, M (Q^1^, Q^3^)	240.00 (198.00, 295.25)	233.00 (196.00, 283.75)	253.50 (208.25, 323.50)	Z=-3.12	0.002
Lymphocyte count, ×10^9^/L, M (Q^1^, Q^3^)	2.48 (1.72, 3.52)	2.45 (1.69, 3.48)	2.56 (1.75, 3.75)	Z=-0.80	0.425
Monocytes count, ×10^9^/L, M (Q^1^, Q^3^)	0.57 (0.43, 0.78)	0.57 (0.43, 0.77)	0.60 (0.44, 0.80)	Z=-1.11	0.266
CRP, mg/l, M (Q^1^, Q^3^)	4.91 (1.67, 11.87)	5.12 (1.70, 11.59)	4.59 (1.46, 14.24)	Z=-0.20	0.840
PCT, ng/ml, M (Q^1^, Q^3^)	0.10 (0.06, 0.21)	0.10 (0.06, 0.20)	0.09 (0.06, 0.22)	Z=-0.46	0.644
NLR, M (Q^1^, Q^3^)	1.30 (0.75, 2.47)	1.25 (0.71, 2.47)	1.35 (0.76, 2.45)	Z=-0.64	0.522
PLR, M (Q^1^, Q^3^)	98.84 (71.90, 134.21)	96.06 (69.97, 132.89)	104.50 (78.32, 141.46)	Z=-1.87	0.062
IBI, M (Q^1^, Q^3^)	6.14 (1.57, 22.36)	6.54 (1.59, 20.86)	5.40 (1.50, 30.24)	Z=-0.15	0.883
PWR, M (Q^1^, Q^3^)	35.93 (27.88, 45.20)	35.93 (28.14, 43.46)	36.13 (27.63, 47.75)	Z=-0.82	0.413
IL-6, ng/ml, M (Q^1^, Q^3^)	10.00 (5.70, 29.30)	10.00 (5.80, 28.10)	9.25 (5.43, 30.45)	Z=-0.08	0.938
ALT, U/L, M (Q^1^, Q^3^)	15.00 (12.00, 19.00)	15.00 (12.00, 19.00)	14.00 (12.00, 19.00)	Z=-0.83	0.406
AST, U/L, M (Q^1^, Q^3^)	39.00 (32.00, 46.00)	40.00 (33.00, 48.00)	37.00 (31.00, 44.00)	Z=-3.21	0.001
TB, μmol/L, M (Q^1^, Q^3^)	5.50 (4.29, 7.20)	5.60 (4.22, 7.27)	5.48 (4.30, 7.00)	Z=-0.52	0.602
Cr, μmol/L, M (Q^1^, Q^3^)	26.00 (21.00, 31.00)	26.00 (22.00, 32.00)	25.00 (20.00, 30.00)	Z=-1.82	0.069
LDH, U/L, M (Q^1^, Q^3^)	346.00 (300.00, 396.75)	347.00 (302.00, 409.00)	344.00 (296.00, 387.00)	Z=-1.26	0.208
BUN,mmol/L, M (Q^1^, Q^3^)	3.67 (3.00, 4.40)	3.70 (2.99, 4.42)	3.65 (3.02, 4.28)	Z=-0.69	0.489
CK, U/L, M (Q^1^, Q^3^)	115.00 (84.25, 155.00)	118.00 (87.00, 157.00)	112.00 (77.00, 149.00)	Z=-1.73	0.083

Z: Mann-Whitney test. WBC, white blood cell count; PLT, platelet count; CRP, C-reactive protein; PCT, procalcitonin; NLR, neutrophil-to-lymphocyte ratio; PLR, platelet-to-lymphocyte ratio; IBI, immune balance index; PWR, platelet-to-white blood cell ratio; IL-6, interleukin-6; ALT, alanine aminotransferase; AST, aspartate aminotransferase; TB, total bilirubin; Cr, creatinine; LDH, lactate dehydrogenase; BUN, blood urea nitrogen; CK, creatine kinase. M, Median, Q^1^, 1st Quartile, Q^3^, 3rd Quartile.

No significant differences were observed in classic inflammatory markers between the two groups, including CRP (4.59 vs. 5.12 mg/L, Z=-0.20, P = 0.840), PCT (0.09 vs. 0.10 ng/mL, Z=-0.46, P = 0.644), and interleukin-6 (IL-6, 9.25 vs. 10.00 ng/mL, Z=-0.08, P = 0.938). Novel inflammatory indices, including NLR, PLR, IBI, and PWR, also exhibited no statistical differences between the two groups (all P>0.05). For organ function markers, AST was statistically lower in the co-detection group (37.0 [31.00--44.00]) compared with the single-detection group (40.0 [33.00--48.00], Z=-3.21, P = 0.001). However, the absolute difference of 3 U/L is clinically negligible, falling within normal physiological variation and below the minimally clinically important difference. Similarly, while PLT was significantly higher in the co-detection group (253.5 vs. 233.0 ×10^9^/L, P = 0.002), the 20.5 ×10^9^/L difference, though statistically significant, is of questionable clinical significance. Alanine aminotransferase, total bilirubin (TB), creatinine (Cr), lactate dehydrogenase (LDH), blood urea nitrogen (BUN), and creatine kinase (CK) showed no between-group differences (all P>0.05).

**Figure 1:**
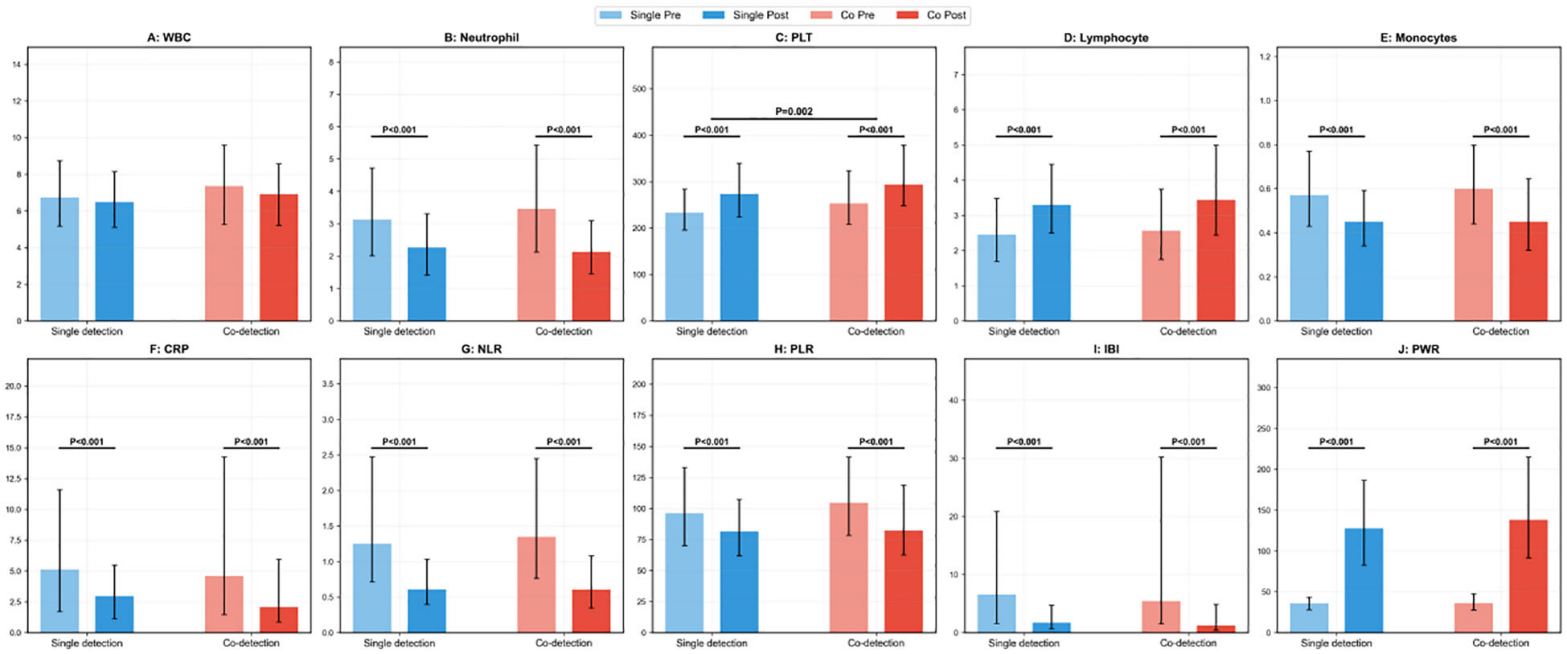
Changes in inflammatory markers before and after treatment in single-detection and co-detection groups. Panels **(A–J)** correspond to WBC, Neutrophil count, PLT, Lymphocyte count, Monocytes count, CRP, NLR, PLR, IBI, and PWR, respectively. Horizontal lines denote P-values for intra-group (pre vs. post-treatment, all P<0.001) and inter-group (single-detection vs. co-detection at pre-treatment, only PLT: P = 0.002) comparisons. Effect sizes (median differences with 95% CI) for pre- vs. post-treatment changes: CRP: -3.2 mg/L (95% CI: -4.1 to -2.3); NLR: -0.45 (95% CI: -0.62 to -0.28); PLR: -15.3 (95% CI: -22.1 to -8.5); IBI: -2.8 (95% CI: -4.5 to -1.1); PWR: +2.1 (95% CI: 0.8 to 3.4); PLT: +28 ×10^9^/L (95% CI: 18 to 38). All effect sizes exceeded the minimally clinically important difference (MCID = 0.5 SD). Abbreviations: WBC, white blood cell count; PLT, platelet count; CRP, C-reactive protein; NLR, neutrophil-to-lymphocyte ratio; PLR, platelet-to-lymphocyte ratio; IBI, immune balance index; PWR, platelet-to-white blood cell ratio;.

Inflammatory markers (WBC, neutrophil, PLT, lymphocyte, monocyte, CRP, NLR, PLR, IBI, PWR) before and after treatment in the two groups were further analyzed (**Figure 1**). Within both the single-detection and co-detection groups, all inflammatory markers showed highly significant differences between pre- and post-treatment values (all P<0.001), with consistent core trends: CRP, NLR, PLR, IBI and PWR were significantly reduced after treatment, whereas PLT was increased. All markers displayed uniform directional changes after treatment in both cohorts.

### 3.3 Co-detection patterns

**Figure 2:**
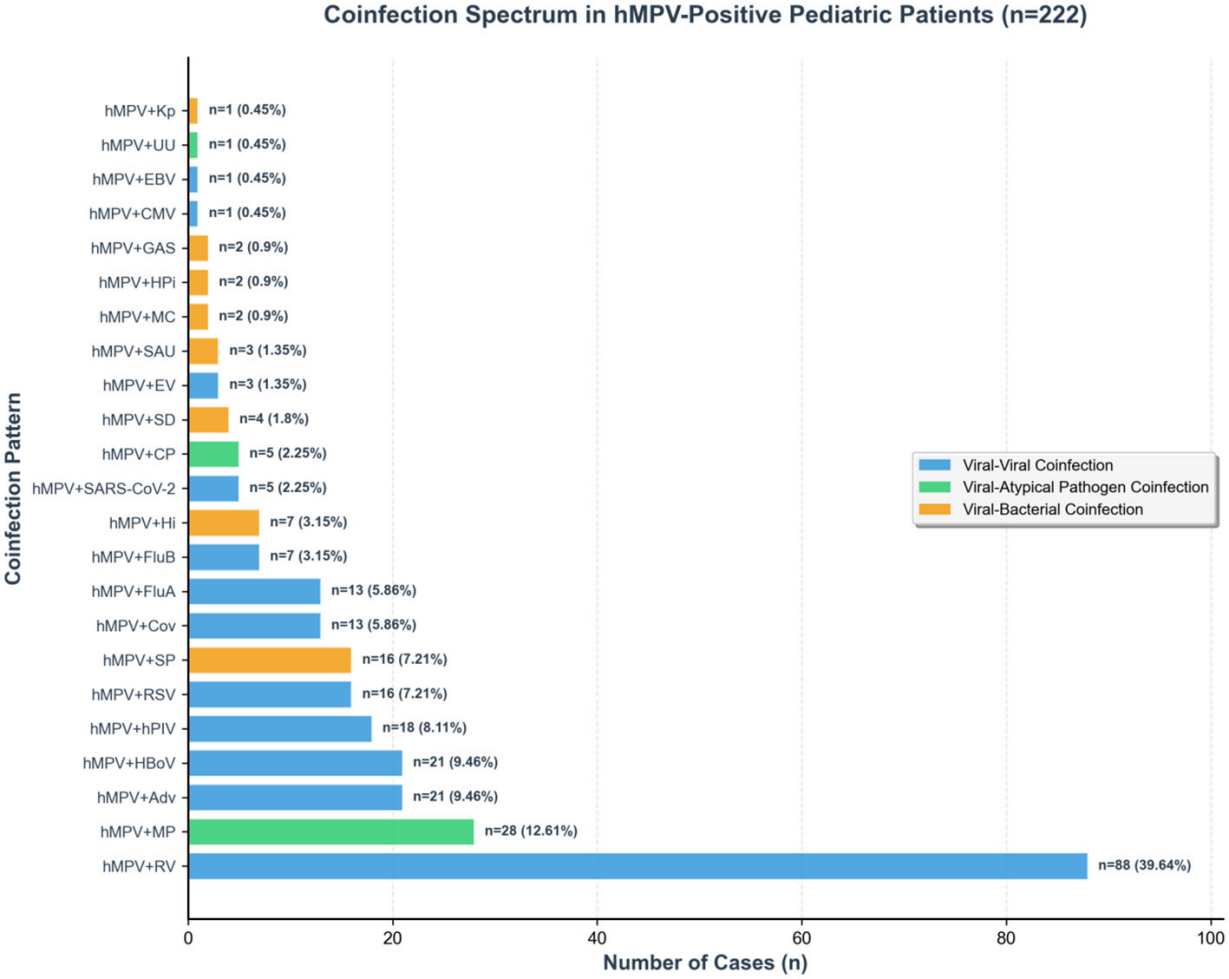
Co-detection spectrum in hMPV-positive pediatric patients. Distribution of co-detection types among 222 hMPV-positive pediatric patients, categorized into viral-viral co-detection (blue), viral-atypical pathogen co-detection (green), and viral-bacterial co-detection (orange). Abbreviations: RV, rhinovirus; AdV, adenovirus; BoV, bocavirus; PIV: parainfluenza virus; RSV, respiratory syncytial virus; M.P *Mycoplasma pneumoniae*; S.P, *Streptococcus pneumoniae*; Hi, *Haemophilus influenzae*; CP, *Chlamydia pneumoniae*.

Among the 222 patients with co-detectable pathogens, viral-viral co-detections were the predominant type (n=153, 68.9%), followed by viral-atypical bacterial/bacterial mixed infections (n=69, 31.1%, **Figure 2**). Rhinovirus (RV) was identified as the most prevalent co-pathogen: hMPV-RV co-detection accounted for 88 cases (39.6% of all co-detections, 57.5% of viral-viral co-detections). Other prevalent viral co-pathogens included adenovirus (AdV, 9.5%), bocavirus (BoV, 9.5%), parainfluenza virus (PIV, 8.1%), and respiratory syncytial virus (RSV, 7.2%).

**Figure 3:**
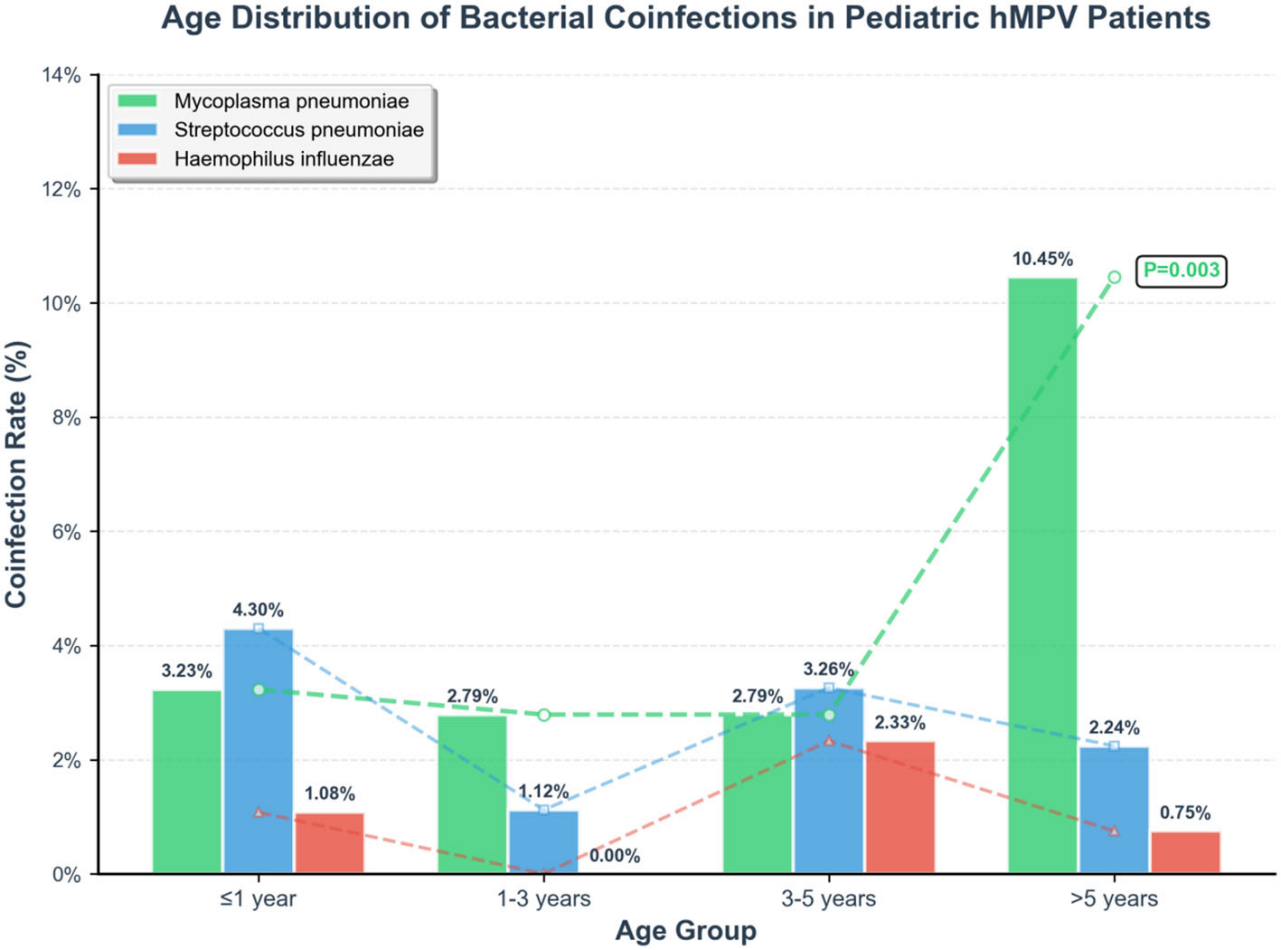
Age-related trends in atypical bacterial and bacterial co-detections among hMPV-positive children. Age distribution of atypical bacterial and bacterial co-detections in pediatric hMPV-positive patients, stratified by three major pathogens: M.P (green), S.P (blue), and *H. influenzae* (red). Percentages of co-detection rates for each pathogen are presented across four age groups (≤1 year, 1–3 years, 3–5 years, >5 years). Significant differences across age groups were observed for M.P (P = 0.003), with a sharp increase in the >5 years age group. Effect sizes (absolute percentage differences with 95% CI) for age-related trends: M. pneumoniae co-detection rate in >5 years vs. ≤1 year: +7.22% (95% CI: 2.1–13.2%, P = 0.003); >5 years vs. 1–3 years: +7.66% (95% CI: 2.1–13.2%, P = 0.003); >5 years vs. 3–5 years: +7.66% (95% CI: 2.1–13.2%, P = 0.003). S.P and H. influenzae showed no significant age-related differences (all P>0.05).

For atypical bacterial/bacterial co-detections, M.P was the most common pathogen (28 cases, 12.6% of all co-detections), followed by S.P (7.2%), *Haemophilus influenzae* (3.2%), and *C. pneumoniae* (2.3%). M.P exhibited a distinct age predilection, with its co-detection rate increasing significantly with age (P = 0.003, **Figure 3**): the co-detection rate was 3.23% in children ≤1 year, 2.79% in 1–3 years, 2.79% in 3–5 years, and 10.45% in children >5 years, indicating a marked predominance in children older than 5 years.

### 3.4 Clinical impact of specific co-detection patterns

**Figure 4:**
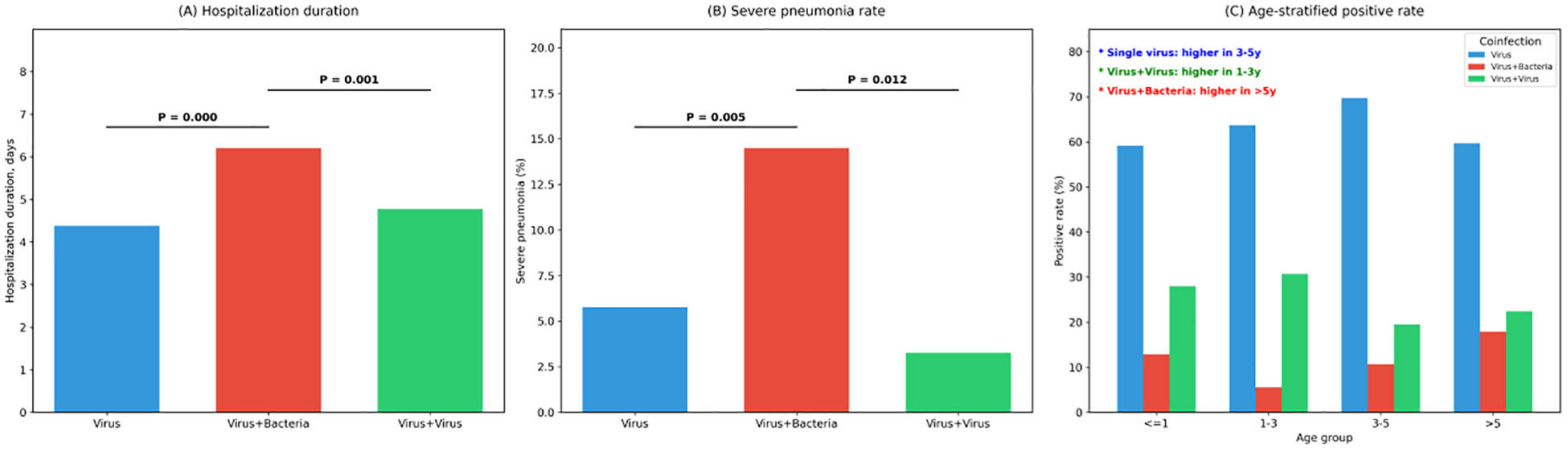
Clinical outcomes and age-stratified positive rates of co-detection patterns in children with hMPV infection. **(A)** Hospitalization duration according to co-detection type with exact P-values for pairwise comparisons. Effect sizes (median differences with 95% CI): viral-bacterial vs. virus: +2.5 days (95% CI: 1.8–3.2, P<0.001); virus-bacteria vs. virus: +2.0 days (95% CI: 1.2–2.8, P = 0.001); virus-virus vs. virus: +0.5 days (95% CI: 0.1–0.9, P = 0.042). **(B)** Proportion of severe pneumonia according to co-detection type. Effect sizes (risk ratios with 95% CI): virus-bacteria vs. virus: RR = 2.50 (95% CI: 1.35–4.62, P = 0.005); virus-bacteria vs. virus-virus: RR = 4.39 (95% CI: 1.68–11.46, P = 0.012); virus-virus vs. virus: RR = 0.57 (95% CI: 0.21–1.54, P = 0.252). Absolute risk differences (ARD): virus-bacteria vs. virus: +8.7% (95% CI: 3.2–14.2%); virus-bacteria vs. virus-virus: +11.2% (95% CI: 4.8–17.6%). **(C)** Age-stratified positive rates of each co-detection pattern across four age groups (≤1 year, 1–3 years, 3–5 years, >5 years). Labels in the upper left indicate subgroups with significantly higher positive rates in the corresponding age groups. co-detection types: Virus (single hMPV infection), Virus+Bacteria (viral-bacterial co-detection), Virus+Virus (virus-viral co-detection). Effect sizes (absolute percentage differences with 95% CI) for key comparisons: Single virus: 3–5 years vs. 1–3 years: +8.31% (95% CI: 2.5–14.1%, P = 0.003); 3–5 years vs. ≤1 year: +12.81% (95% CI: 11.2–15.4%, P<0.001). Virus-viral co-detection: 1–3 years vs. >5 years: +6.05% (95% CI: 1.2–10.9%, P = 0.011). 1–3 years vs. 3–5 years: +9.05% (95% CI: 8.2–10.9%, P = 0.011). Virus-bacterial co-detection: >5 years vs. ≤1 year: +6.66% (95% CI: 2.1–13.2%, P = 0.003); >5 years vs. 3–5 years: +7.16% (95% CI: 6.3–13.2%, P = 0.003); >5 years vs. 1–3 years: +11.41% (95% CI: 0.8–10.0%, P = 0.018).

Hospitalization duration differed significantly among the three groups (F = 23.662, P<0.001): the median hospitalization duration was 4.0 days in the single-virus group, 4.5 days in the virus-viral co-detection group, and 6.5 days in the viral-bacterial co-detection group, indicating that viral-bacterial co-detection was associated with significantly longer hospital stays compared with both single virus infection and viral-viral co-detection (P = 0.000 and P = 0.001, respectively, **Figure 4A**).

The proportion of severe pneumonia also differed significantly among the three groups (χ²=10.674, P = 0.005), with the highest rate observed in the viral-bacterial co-detection group (14.5%, 10/69), followed by the single virus infection group (5.8%, 23/399) and the virus-viral co-detection group (3.3%, 5/153); pairwise comparison showed a significant difference between viral-bacterial co-detection and single virus infection (P = 0.005), and between viral-bacterial co-detection and virus-viral co-detection (P = 0.012) (**Figure 4B**).

Age-stratified analysis demonstrated distinct distribution characteristics of co-detection patterns across different age groups: single virus infection had a higher positive rate in children aged 3–5 years, virus-viral co-detection had a higher positive rate in children aged 1–3 years, and viral-bacterial co-detection had a higher positive rate in children older than 5 years compared with other age groups (**Figure 4C**).

### 3.5 Risk factors for clinical prognosis

Univariate and multivariate logistic regression analyses were performed to identify independent risk factors associated with severe pneumonia in children with hMPV infection (**Table 3**). In univariate analysis, viral-bacteria mixed infection, infection with S.P, prolonged fever duration, elevated CRP (>20 mg/L), and elevated PCT (>0.5 ng/ml) were significantly associated with increased risk of severe pneumonia, whereas older age (age >5 years) was associated with reduced risk (all P < 0.05).

**Table 3:** Independent risk factors for severe pneumonia in children with hMPV infection identified by multivariate logistic regression.

Variables	Univariate analysis					Multivariate analysis				
	β	S.E	Z	*P*	OR (95%CI)	β	S.E	Z	*P*	OR (95%CI)
Mixed infection										
hMPV					1.00 (Reference)					1.00 (Reference)
Virus+Bacteria	1.02	0.40	2.52	0.012	2.77 (1.26 - 6.11)	-0.19	0.70	-0.27	0.785	0.83 (0.21 - 3.23)
Virus+Virus	-0.59	0.50	-1.18	0.238	0.55 (0.21 - 1.48)	-0.60	0.52	-1.15	0.252	0.55 (0.20 - 1.53)
Streptococcus pneumoniae										
Negative					1.00 (Reference)					1.00 (Reference)
Positive	2.67	0.54	4.97	<0.001	14.40 (5.03 - 41.23)	2.13	0.88	2.42	0.015	8.43 (1.50 - 47.32)
Fever duration, days	0.14	0.06	2.20	0.028	1.15 (1.02 - 1.29)	0.17	0.08	2.24	0.025	1.19 (1.02 - 1.38)
Age										
≤1					1.00 (Reference)					1.00 (Reference)
>5	-2.77	1.06	-2.62	0.009	0.06 (0.01 - 0.50)	-3.57	1.11	-3.21	0.001	0.03 (0.00 - 0.25)
1-3	-0.43	0.44	-0.98	0.329	0.65 (0.27 - 1.54)	-0.93	0.52	-1.78	0.075	0.40 (0.14 - 1.10)
3-5	-0.55	0.43	-1.26	0.207	0.58 (0.25 - 1.35)	-1.61	0.55	-2.92	0.004	0.20 (0.07 - 0.59)
Gender										
Boy					1.00 (Reference)					
Girl	0.29	0.34	0.88	0.380	1.34 (0.70 - 2.59)					
Infection Type										
Single detection					1.00 (Reference)					
Co- detection	0.17	0.34	0.49	0.621	1.18 (0.60 - 2.32)					
CRP>20 mg/l										
No					1.00 (Reference)					1.00 (Reference)
Yes	1.23	0.36	3.37	<0.001	3.41 (1.67 - 6.96)	0.71	0.46	1.55	0.121	2.03 (0.83 - 4.96)
PCT>0.5 ng/ml										
No					1.00 (Reference)					1.00 (Reference)
Yes	1.76	0.36	4.83	<0.001	5.80 (2.84 - 11.81)	1.45	0.45	3.22	0.001	4.27 (1.76 - 10.33)

The wide confidence interval for S.P co-detection (aOR=8.43, 95% CI: 1.50–47.32) reflects the small number of events (n=7 S. pneumoniae co-detections, with only 2 progressing to severe pneumonia). Sensitivity analysis excluding S.P co-detection showed that PCT ≥0.5 ng/mL (aOR=4.15, 95% CI: 1.72–10.01) and fever duration (aOR=1.18, 95% CI: 1.01–1.37) remained significant independent predictors. Abbreviations: SE, standard error; OR, odds ratio; CI, confidence interval; CRP, C-reactive protein; PCT, procalcitonin.

After adjusting for potential confounders in the multivariate model, four factors remained independently associated with disease severity. Specifically, infection with S.P (aOR = 8.43, 95% CI: 1.50–47.32, P = 0.015), prolonged fever duration (aOR = 1.19, 95% CI: 1.02–1.38, P = 0.025), and elevated PCT (>0.5 ng/ml, aOR = 4.27, 95% CI: 1.76–10.33, P = 0.001) were independent risk factors for severe pneumonia. In contrast, older age groups showed significant protective effects: children aged >5 years (aOR = 0.03, 95% CI: 0.00–0.25, P = 0.001) and those aged 3–5 years (aOR = 0.20, 95% CI: 0.07–0.59, P = 0.004) had substantially lower odds of developing severe pneumonia compared with children aged ≤1 year. These findings indicate that age, bacterial co-detection with S.P, fever duration, and PCT level are key prognostic indicators in pediatric hMPV infection (**Supplementary Figure 1**). The multivariate model demonstrated good goodness-of-fit (Hosmer-Lemeshow test: χ²=6.42, P = 0.601) and satisfactory discriminatory ability (C-statistic: 0.847, 95% CI: 0.782-0.912; Nagelkerke R²=0.312). Multicollinearity assessment showed all VIF values <2.0 (range: 1.05-1.78), indicating no significant multicollinearity among predictors.

ROC analysis was performed to evaluate the discriminatory performance of PCT for severe pneumonia prediction. PCT demonstrated moderate discriminatory ability (AUC = 0.742, 95% CI: 0.668–0.816). The optimal cutoff by Youden index was 0.48 ng/mL (sensitivity: 68.4%, specificity: 76.2%). At the clinically used ≥0.5 ng/mL threshold, sensitivity was 65.8%, specificity 78.4%, positive predictive value 15.2%, and negative predictive value 97.8% (**Supplementary Figure 2**). Novel inflammatory indices (NLR, PLR, IBI, PWR) showed no significant association with severe pneumonia in univariate analysis (NLR: OR = 1.12, P = 0.342; PLR: OR = 1.01, P = 0.876; IBI: OR = 0.98, P = 0.765; PWR: OR = 0.95, P = 0.612) and did not improve model performance when added to the multivariate model (ΔAUC <0.01 for all indices). Restricted cubic spline analysis confirmed the linear relationship between fever duration and severe pneumonia risk (non-linearity test P = 0.342).

## 4 Discussion

This retrospective study systematically analyzed the clinical characteristics, co-detection patterns, laboratory features, and prognostic risk factors of 621 pediatric patients with laboratory-detected hMPV infection, providing a comprehensive overview of the clinical spectrum of hMPV infection in children. Our findings revealed that hMPV infection is most prevalent in preschool children (3–5 years), with viral-viral co-detection as the dominant co-detection type and rhinovirus as the most common co-pathogen. Notably, viral-bacterial co-detection was associated with prolonged hospitalization and an increased risk of severe pneumonia; multivariate logistic regression further identified S.P co-detection, prolonged fever duration, and elevated PCT ≥0.5 ng/mL were independently associated with increased risk for severe pneumonia in hMPV-infected children, while older age groups (3–5 years and >5 years) were associated with reduced risk. These results enrich the clinical understanding of pediatric hMPV infection and provide evidence-based insights for clinical risk stratification, targeted etiological screening, and personalized intervention in children with hMPV infection.

Consistent with previous studies, the present study found that hMPV infection primarily affects preschool children, with a median age of 3.50 years and the highest prevalence in the 3–5 years age group (Li et al., 2025). This age distribution may be attributed to the immature immune system of preschool children and increased exposure to pathogens in collective settings (e.g., kindergartens), which increases their susceptibility to respiratory viral infections (Yang et al., 2025). No significant differences in age and sex ratios were observed between the hMPV single-detection and co-detection groups, suggesting that age and sex may not be associated with the risk of co-detection in hMPV-infected children, which is in line with the findings of a multicenter study in Southern China (Zhang et al., 2018). In terms of clinical symptoms, cough was the most common manifestation (97.4%), while wheezing and dyspnea were less frequent, indicating that hMPV infection is mainly characterized by mild to moderate respiratory symptoms in most children, which is consistent with the clinical features of hMPV infection reported in the Chinese pediatric population (Wang et al., 2021).

A key finding of this study is that 35.7% of hMPV-infected children had co-detectable pathogens, with viral-viral co-detection (68.9%) predominating over viral-bacterial co-detection (31.1%), and rhinovirus was the most prevalent co-pathogen (39.6% of all co-detections). This is consistent with previous reports that respiratory viruses are the most common co-pathogens in hMPV infection, and rhinovirus is the leading coinfecting virus (Yang et al., 2025). The high co-detection rate of hMPV and rhinovirus may be attributed to their shared transmission routes (respiratory droplets) and overlapping seasonal prevalence, which increases the likelihood of simultaneous or sequential infection (Jobe et al., 2025). For bacterial co-detections, S.P was identified as the key pathogenic bacterium associated with severe hMPV infection in the subsequent prognostic analysis (Smyrnaios et al., 2025). Age-stratified analysis of co-detection patterns revealed that single virus infection was most common in children aged 3–5 years, virus-viral co-detection in those aged 1–3 years, and viral-bacterial co-detection in children >5 years (Fu et al., 2024). This age-specific distribution of co-detection patterns provides a valuable reference for age-based clinical etiological screening and targeted testing in pediatric hMPV infection.

Regarding laboratory findings, baseline PLT and AST showed statistically significant but clinically negligible differences between Single-detection and co-detection groups (20.5 ×10^9^/L and 3 U/L differences, respectively). No significant differences were observed in classic inflammatory markers (CRP, PCT, IL-6) at baseline. The lack of meaningful baseline differences in inflammatory markers between single-detection and co-detection groups suggests limited diagnostic utility for etiological discrimination at admission, consistent with observations in other respiratory viral infections where inflammatory responses are relatively muted (Adams et al., 2015; He et al., 2025). Notably, all inflammatory markers demonstrated significant improvements after treatment in both groups, with consistent directional trends (reduction of CRP, NLR, PLR, IBI, PWR and increase of PLT), confirming the effectiveness of conventional anti-infective and symptomatic treatments for hMPV infection regardless of co-detection status. This finding also indicates that baseline inflammatory markers have limited discriminatory value in distinguishing hMPV single-detection from co-detection, which differs from observations in influenza virus infection (Adams et al., 2015), possibly due to the relatively milder inflammatory response induced by hMPV compared with other respiratory viruses. However, the subsequent prognostic analysis further underscored the high clinical utility of PCT in assessing hMPV infection severity, thereby compensating for the limited discriminatory capacity of baseline inflammatory markers in co-detection identification (Sartori et al., 2021). It is important to distinguish between the diagnostic utility of baseline inflammatory markers (which showed limited ability to differentiate Single-detection from co-detection) and their prognostic value (where PCT ≥0.5 ng/mL emerged as a strong independent association with severe pneumonia). This distinction reflects different clinical applications: etiological diagnosis versus severity prediction.

The clinical impact analysis of co-detection patterns demonstrated that hospitalization duration differed significantly among the single virus, virus-viral co-detection, and viral-bacterial co-detection groups, with viral-bacterial co-detection associated with the longest hospitalization. Scotta et al. further confirmed in their systematic review that viral co-detections in children are associated with increased disease severity and longer hospital stays (Scotta et al., 2016). Furthermore, the proportion of severe pneumonia was significantly higher in the viral-bacterial co-detection group (14.5%) compared with the other two groups, indicating that viral-bacterial co-detection is a pivotal factor associated with severe clinical outcomes in hMPV-infected children. This is consistent with findings by Zhou et al., who reported that invasive pneumococcal pneumonia frequently co-occurs with respiratory viral infections, leading to more severe clinical presentations (Zhou et al., 2012). Several mechanisms may underlie this phenomenon (1): bacterial co-detection can exacerbate the respiratory tract inflammatory response, resulting in severe lung tissue damage and impaired gas exchange (Sumitomo and Kawabata, 2024, 2); hMPV infection can compromise the respiratory mucosal barrier, disrupting the body’s first line of defense and increasing susceptibility to secondary bacterial infection (Pandey et al., 2026, 3); the synergistic interaction between virus and bacteria can suppress host innate and adaptive immune responses, leading to persistent infection and disease progression (Sun et al., 2026). Experimental studies have demonstrated that prior hMPV infection predisposes to severe pneumococcal pneumonia, supporting the clinical observation of viral-bacterial synergy (Papenburg et al., 2012). In contrast, virus-viral co-detection was not associated with a significant increase in severe pneumonia rate (3.3%), suggesting that the nature of the coinfecting pathogen (bacterial vs. viral) is a critical determinant of hMPV infection severity (Mao and Wu, 2024). This distinction is corroborated by prospective cohort studies showing that while viral co-detections are common in hMPV-infected children, which are not independently associated with increased disease severity, whereas bacterial co-detections significantly worsen outcomes (Asner et al., 2015). This represents an important clinical finding of our study and is further corroborated by the subsequent regression analysis identifying S.P co-detection as an independent risk factor for severe pneumonia.

Multivariate logistic regression analysis, after adjusting for potential confounders and screening through univariate analysis, identified three core independent risk factors and two protective factors for severe pneumonia. Specifically, S.P co-detection (aOR=8.43, 95%CI:1.50–47.32, P = 0.015) emerged as the strongest independent risk factor, underscoring the pivotal role of S.P in the progression of hMPV infection to severe pneumonia. In a recent retrospective cohort study of 203 children with pneumococcal pneumonia, S.P co-detection was independently associated with more severe disease outcomes, particularly when combined with atypical pathogens (Bao et al., 2026). The underlying mechanisms may involve the synergistic pathogenic effects of hMPV and S.P on the respiratory system: hMPV infection can induce airway epithelial cell damage and local immune dysfunction, while S.P can adhere to and invade the compromised respiratory mucosa, produce pneumolysin and other virulence factors, and trigger an exaggerated systemic inflammatory response, thereby exacerbating lung inflammation, airway obstruction, and alveolar damage (Chen et al., 2021). Prolonged fever duration (aOR=1.19, 95%CI:1.02–1.38, P = 0.025) was also confirmed as an independent risk factor, with each additional day of fever associated with a 19% increase in the risk of severe pneumonia. Persistent fever in hMPV infection reflects a sustained inflammatory response, which may indicate inadequate host immune clearance of the pathogen or underlying bacterial co-detection, serving as a readily accessible clinical indicator for identifying children at high risk of severe disease (Nijman et al., 2023). Elevated PCT ≥0.5 ng/mL (aOR=4.27, 95%CI:1.76–10.33, P = 0.001) represented another key independent risk factor, consistent with the established role of PCT as a sensitive and specific biomarker for bacterial infection and systemic inflammation (Tissières et al., 2025). PCT elevation in hMPV-infected children serves as a reliable indicator of bacterial co-detection (particularly S.P) or severe inflammatory response, and its predictive value for severe pneumonia surpassed that of CRP (which lost statistical significance in multivariate analysis), rendering it an optimal laboratory parameter for clinical risk stratification (Wei et al., 2023; Zhang et al., 2024). Notably, this study found that older age groups (3–5 years and >5 years) were independent protective factors against severe pneumonia in hMPV-infected children, with children aged >5 years exhibiting the most pronounced protective effect (aOR=0.03, P = 0.001) and those aged 3–5 years also demonstrating significant protection (aOR=0.20, P = 0.004) compared with infants aged ≤1 year. This is consistent with epidemiological evidence showing that hMPV-associated ALRIs are significantly more common in children under 1 year (Hacker et al., 2022). The immaturity of the infant immune system, characterized by waning maternal antibodies, a relative antibody nadir from 3-12 months, and incomplete cell-mediated immunity until 12 months of age, combined with underdeveloped mucosal barrier function, contributes to this increased vulnerability (Palmer et al., 2024). In contrast, children aged 3 years and above have a more mature immune system, enhanced pathogen clearance capacity, and better-developed respiratory tract function, which collectively mitigate the risk of severe pneumonia following hMPV infection (Mao et al., 2026). This finding provides a robust clinical rationale for prioritizing intensive monitoring and early intervention in infants aged ≤1 year with hMPV infection.

Our findings have several clinical implications. First, age-based risk stratification should be implemented: infants ≤1 year require intensive monitoring given their substantially higher risk of severe pneumonia (reference group in our model). Second, PCT-guided management may be valuable: children with hMPV infection and PCT ≥0.5 ng/mL should prompt screening for bacterial co-detection (particularly S.P) and consideration of empirical antibiotic therapy. The high negative predictive value (97.8%) at this threshold supports its utility in ruling out severe bacterial co-detection. Third, targeted testing strategies: children >5 years with hMPV should be screened for M.P given its age-related predilection. Fourth, duration of fever monitoring: persistent fever beyond 3 days should raise concern for severe disease progression and prompt reassessment. These implications should be validated in prospective studies before implementation in clinical practice.

This study has several strengths: first, it included a relatively large sample size of 621 pediatric patients, thereby reducing selection bias and enhancing the reliability and statistical power of the findings; second, it systematically analyzed co-detection patterns and their age distribution characteristics, elucidating the clinical impact of different co-detection patterns on hospitalization duration and disease severity; third, it employed rigorous univariate and multivariate logistic regression analyses with adjustment for potential confounders, identifying independent risk and protective factors for severe hMPV infection with substantial clinical relevance; fourth, the study confirmed the predictive value of PCT and fever duration for severe pneumonia, and identified S.P co-detection as the core bacterial risk factor, providing clear targets for targeted etiological testing and anti-infective treatment in clinical practice. However, this study has several limitations that should be acknowledged. First, as a single-center retrospective study, our findings may not be fully generalizable to other pediatric populations with distinct regional epidemiological characteristics. The relatively mild disease spectrum in our cohort (6.1% severe pneumonia, 1.61% PICU admission, zero mortality) limits generalizability to critically ill populations. Second, important potential confounders including prior antibiotic use, vaccination status (particularly pneumococcal conjugate vaccine), and detailed comorbidity histories were not consistently available in retrospective records. Third, the wide confidence interval for S.P co-detection (aOR=8.43, 95% CI: 1.50–47.32) should be interpreted with caution due to the small number of events (n=7 co-detections, 2 severe cases), which may indicate overfitting. Fourth, we did not apply formal multiple comparison corrections for exploratory analyses (inflammatory markers, co-detection patterns), which may increase the risk of Type I error; these findings should be considered hypothesis-generating rather than definitive. Fifth, the study did not collect data on seasonal prevalence of hMPV infection or analyze different hMPV genotypes, which may influence disease severity and co-detection patterns. Sixth, despite lack of external funding, diagnostic quality was maintained through standardized protocols in laboratory; however, resource constraints limited our ability to perform whole-genome sequencing for hMPV genotyping. Finally, as a retrospective observational study, our findings demonstrate associations rather than causal relationships. Future multicenter prospective studies are needed to validate these findings and establish causality.

## 5 Conclusion

This retrospective study demonstrates that hMPV infection is most prevalent among preschool children, with viral-viral co-detection being the predominant form of co-detection. Viral-bacterial co-detection was associated with significantly longer hospital stays and higher rates of severe pneumonia. Co-infection with S.P, prolonged fever duration, and a PCT level of ≥ 0.5 ng/mL were independently associated with increased risk of severe pneumonia; increasing age (especially over 3 years) was associated with reduced risk. Clinicians should remain highly vigilant for infants aged 1 year or younger. For children with persistent fever or elevated PCT, early screening for S.P should be carried, and empirical antibacterial treatment may be warranted while strengthening monitoring. These associations require validation in future multicenter prospective studies before implementation in clinical practice.

## Data Availability

The original contributions presented in the study are included in the article/**Supplementary Material**. Further inquiries can be directed to the corresponding authors.

## References

[B1] Adams O. Weis J. Jasinska K. Vogel M. Tenenbaum T. (2015). Comparison of human metapneumovirus, respiratory syncytial virus and rhinovirus respiratory tract infections in young children admitted to hospital. J. Med. Virol. 87, 275–280. doi: 10.1002/jmv.24025 25074284 PMC7166420

[B2] Amarin J. Z. Toepfer A. P. Spieker A. J. Hayek H. Stopczynski T. Qwaider Y. Z. . (2026). Respiratory syncytial virus co-detection with other respiratory viruses is not significantly associated with worse clinical outcomes among children aged <2 years: New Vaccine Surveillance Network, 2016-2020. Clin. Infect. Dis. 82, 358–365. doi: 10.1093/cid/ciaf194 40341868

[B3] Aradhana Mishra V. Rup A. R. Mohakud N. K. (2025). Severe manifestations of human metapneumovirus with co-infections: a case series and literature review. Cureus 17, e99262. doi: 10.7759/cureus.99262 41552058 PMC12806568

[B4] Asner S. A. Rose W. Petrich A. Richardson S. Tran D. J. (2015). Is virus co-detection a predictor of severity in children with viral respiratory infections? Clin. Microbiol. Infect. 21, 264.e1–264.e6. doi: 10.1016/j.cmi.2014.08.024 25596778 PMC7128494

[B5] Bao M. Wang S. Yi Y. Ni T. (2026). Risk factors affecting severe pneumococcal pneumonia in children: a retrospective study. BMC Infect. Dis. 26 (1), 441. doi: 10.1186/s12879-026-12716-w 41612220 PMC12924381

[B6] Bhattacharya S. Bhattacharjee S. Singh A. (2025). Human metapneumovirus: a comprehensive epidemiological analysis of a global respiratory threat. Infect. Chemother. 57, 194–202. doi: 10.3947/ic.2025.0019 40343424 PMC12230387

[B7] Chen Y. Y. Huang C. T. Li S. W. Pan Y. J. Lin T. L. Huang Y. Y. . (2021). Bacterial factors required for Streptococcus pneumoniae co-detection with influenza A virus. J. Biomed. Sci. 28, 60. doi: 10.1186/s12929-021-00756-0 34452635 PMC8395381

[B8] Ding Y. Jing C. Wei J. Wang D. Li W. Wang M. . (2025). Comparison of the diagnostic capabilities of tNGS and mNGS for pathogens causing lower respiratory tract infections: a prospective observational study. Front. Cell. Infect. Microbiol. 15, 1578939. doi: 10.3389/fcimb.2025.1578939 40557317 PMC12185458

[B9] Evelyn O. Jaime F. S. David M. Lorena A. Jenifer A. Oscar G. (2019). Prevalence, clinical outcomes and rainfall association of acute respiratory infection by human metapneumovirus in children in Bogotá, Colombia. BMC Pediatr. 19, 345. doi: 10.1186/s12887-019-1734-x 31601181 PMC6785857

[B10] Fu C. Zhou C. Zheng C. Li S. Song W. Yao J. . (2024). Etiological analysis of acute respiratory infections in hospitalized children after the relaxation of COVID-19 non-pharmacological interventions in Quzhou, China. BMC Infect. Dis. 24, 1362. doi: 10.1186/s12879-024-10257-8 39609752 PMC11603909

[B11] Gnanasekaran S. Bashar M. A. Rajan A. K. Prabhat P. (2025). Emerging threat of human metapneumovirus (HMPV) and strategies for its containment and control. Infect. Genet. Evol. 131, 105758. doi: 10.1016/j.meegid.2025.105758 40345565

[B12] Gu D. Liu J. Wang J. She J. Lu B. (2025). Integrating DNA and RNA sequencing for enhanced pathogen detection in respiratory infections. J. Transl. Med. 23, 325. doi: 10.1186/s12967-025-06342-4 40087699 PMC11907987

[B13] Hacker K. Kuan G. Vydiswaran N. Chowell-Puente G. Patel M. Sanchez N. . (2022). Pediatric burden and seasonality of human metapneumovirus over 5 years in Managua, Nicaragua. Influenza Other Respir. Viruses 16, 1112–1121. doi: 10.1111/irv.13034 35965382 PMC9530515

[B14] He P. Hu F. Wang F. (2025). A study of the relationship between cough and wheezing complicated by common respiratory viral infections in infants and secondary thrombocythemia. PloS One 20, e0326369. doi: 10.1371/journal.pone.0326369 40632767 PMC12240307

[B15] Howard L. M. Edwards K. M. Zhu Y. Grijalva C. G. Self W. H. Jain S. . (2021). Clinical features of human metapneumovirus-associated community-acquired pneumonia hospitalizations. Clin. Infect. Dis. 72, 108–117. doi: 10.1093/cid/ciaa088 32010955 PMC7823075

[B16] Jobe N. B. Rose E. Winn A. K. Goldstein L. Schneider Z. D. Silk B. J. (2025). Human metapneumovirus seasonality and co-circulation with respiratory syncytial virus - United States, 2014-2024. MMWR Morb Mortal Wkly Rep. 74, 182–187. doi: 10.15585/mmwr.mm7411a1 40179043 PMC11970723

[B17] Khales P. Razizadeh M. H. Ghorbani S. Tameshkel F. S. Saadati H. Vazirzadeh M. . (2025). The prevalence and role of human metapneumovirus in respiratory tract infections: a systematic review and meta-analysis of global data. EClinicalMedicine 88, 103480. doi: 10.1016/j.eclinm.2025.103480 40932845 PMC12418874

[B18] Kikuchi S. Trimaille A. Carmona A. Vu M. Granier A. Truong D. . (2025). Inflammatory burden index and mortality following transcatheter aortic valve replacement. Eur. Heart J. 46, ehaf784.2380. doi: 10.1093/eurheartj/ehaf784.2380

[B19] Li A. Gong C. Wang L. Han Y. Kang L. Hu G. . (2025). Epidemiological and phylogenetic characteristics of human metapneumovirus in Beijing, China, 2014-2024. Signal. Transduct Target Ther. 10, 300. doi: 10.1038/s41392-025-02377-7 40921736 PMC12417542

[B20] Li Y. Min L. Zhang X. (2021). Usefulness of procalcitonin (PCT), C-reactive protein (CRP), and white blood cell (WBC) levels in the differential diagnosis of acute bacterial, viral, and mycoplasmal respiratory tract infections in children. BMC Pulm. Med. 21, 386. doi: 10.1186/s12890-021-01756-4 34836530 PMC8620633

[B21] Mao B. Chen D. Li Z. Lu W. Liu W. (2026). Epidemiological characteristics of hospitalized pediatric patients with acute respiratory tract infection during and after the COVID-19 period in Ningbo, China: a retrospective single-center study. BMC Infect. Dis. doi: 10.1186/s12879-026-13316-4 42177435 PMC13383466

[B22] Mao S. Wu L. (2024). Co-detection of viruses in children with community-acquired pneumonia. BMC Pediatr. 24, 457. doi: 10.1186/s12887-024-04939-0 39014398 PMC11250944

[B23] Nijman R. G. Tan C. D. Hagedoorn N. N. Nieboer D. Herberg J. A. Balode A. . (2023). Are children with prolonged fever at a higher risk for serious illness? A prospective observational study. Arch. Dis. Child 108, 632–639. doi: 10.1136/archdischild-2023-325343 37185174

[B24] Palmer A. C. Bedsaul-Fryer J. R. Stephensen C. B. (2024). Interactions of nutrition and infection: the role of micronutrient deficiencies in the immune response to pathogens and implications for child health. Annu. Rev. Nutr. 44, 99–124. doi: 10.1146/annurev-nutr-062122-014910 38724105

[B25] Pandey V. Shahi P. Kolios G. Uddin M. I. Spathakis M. Collins A. R. . (2026). Human metapneumovirus (hMPV): the virus who came with the common cold. Infection 54, 1–13. doi: 10.1007/s15010-025-02626-5 40794271 PMC12864362

[B26] Pantea M. Iacob D. Dima M. Prodan M. Belei O. Negrean R. A. . (2024). Predictive value of inflammatory markers NLR, PLR, APRI, SII, and liver function tests in systemic inflammatory response syndrome detection in full-term newborns. Children (Basel) 11, 593. doi: 10.3390/children11050593 38790588 PMC11119895

[B27] Papenburg J. Hamelin M.È. Ouhoummane N. Carbonneau J. Ouakki M. Raymond F. . (2012). Comparison of risk factors for human metapneumovirus and respiratory syncytial virus disease severity in young children. J. Infect. Dis. 206, 178–189. doi: 10.1093/infdis/jis333 22551815 PMC7114627

[B28] Ribó-Molina P. van Nieuwkoop S. Mykytyn A. Z. van Run P. Lamers M. M. Haagmans B. L. . (2024). Human metapneumovirus infection of organoid-derived human bronchial epithelium represents cell tropism and cytopathology as observed in *in vivo* models. mSphere 9, e0074323. doi: 10.1128/msphere.00743-23 38265200 PMC10900881

[B29] Sartori L. Zhu Y. Grijalva C. Ampofo K. Gesteland P. Johnson J. . (2021). Pneumonia severity in children: utility of procalcitonin in risk stratification. Hosp. Pediatr. 11, 215–222. doi: 10.1542/hpeds.2020-001842 33579748 PMC7898232

[B30] Scotta M. C. Chakr V. C. de Moura A. Becker R. G. de Souza A. P. Jones M. H. . (2016). Respiratory viral co-detection and disease severity in children: a systematic review and meta-analysis. J. Clin. Virol. 80, 45–56. doi: 10.1016/j.jcv.2016.04.019 27155055 PMC7185664

[B31] Smyrnaios A. Krokstad S. Follestad T. Christensen A. Risnes K. Nordbø S. A. . (2025). The significance of upper airway density of Streptococcus pneumoniae and respiratory viruses in the aetiology and severity of paediatric community-acquired pneumonia in Norway: an observational study. J. Microbiol. Immunol. Infect. 59 (3), 358–363. doi: 10.1016/j.jmii.2025.08.019 40897650

[B32] Sumitomo T. Kawabata S. (2024). Respiratory tract barrier dysfunction in viral-bacterial co-infection cases. Jpn. Dent. Sci. Rev. 60, 44–52. doi: 10.1016/j.jdsr.2023.12.006 38274948 PMC10808858

[B33] Sun Y. Shao W. Wang X. Yang R. Li W. Xu C. . (2026). Epidemiology and co-infection networks of pediatric respiratory pathogens in eastern China after COVID-19 restriction relaxation: a retrospective study. Front. Cell. Infect. Microbiol. 16, 1795806. doi: 10.3389/fcimb.2026.1795806 42052093 PMC13110959

[B34] The Subspecialty Group of Respiratory, the Society of Pediatrics, Chinese Medical Association (2024). Guidelines for the management of community-acquired pneumonia in children (2024 revision). Chin. J. Pediatr. 62, 920–930. doi: 10.3760/cma.j.cn112140-20240728-00523 39327958

[B35] Tissières P. Esteban Torné E. Hübner J. Randolph A. G. Rey Galán C. Weiss S. L. (2025). Use of procalcitonin in therapeutic decisions in the pediatric intensive care unit. Ann. Intensive Care 15, 55. doi: 10.1186/s13613-025-01470-y 40268774 PMC12018671

[B36] Üçsular F. Polat G. Karadeniz G. Ayranci A. Keskin M. Buyukşirin M. . (2020). Predictive value of platelet-to-lymphocyte ratio and neutrophil-to-lymphocyte ratio in patients with hypersensitivity pneumonia. Sarcoidosis Vasc. Diffuse Lung Dis. 37, e2020012. doi: 10.36141/svdld.v37i4.9966 33597799 PMC7883515

[B37] Van den Hoogen B. G. de Jong J. C. Groen J. Kuiken T. de Groot R. Fouchier R. A. . (2001). A newly discovered human pneumovirus isolated from young children with respiratory tract disease. Nat. Med. 7, 719–724. doi: 10.1038/89098 11385510 PMC7095854

[B38] Wang C. Wei T. Ma F. Wang H. Guo J. Chen A. . (2021). Epidemiology and genotypic diversity of human metapneumovirus in paediatric patients with acute respiratory infection in Beijing, China. Virol. J. 18 (1), 40. doi: 10.1186/s12985-021-01508-0 33602245 PMC7890387

[B39] Wang F. Han A. Hong W. Mao Y. Sun W. Hu Y. . (2026). Comparison of targeted next-generation sequencing and conventional tests for pathogen detection in community-acquired and severe community-acquired pneumonia: a retrospective cohort study. Front. Microbiol. 17, 1799720. doi: 10.3389/fmicb.2026.1799720 42199547 PMC13200559

[B40] Wei S. Wang L. Lin L. Liu X. (2023). Predictive values of procalcitonin for co-detections in patients with COVID-19: a systematic review and meta-analysis. Virol. J. 20, 92. doi: 10.1186/s12985-023-02042-x 37158904 PMC10166029

[B41] WHO (2013). Pocket Book of Hospital Care for Children: Guidelines for the Management of Common Childhood Illnesses (Geneva: World Health Organization). 24006557

[B42] Yang F. Jiang L. Cao Q. Yi M. Zhao Q. (2025). Evaluation of targeted next-generation sequencing for microbiological diagnosis of acute lower respiratory infection. Front. Microbiol. 16, 1615965. doi: 10.3389/fmicb.2025.1615965 40901079 PMC12399591

[B43] Yang S. Lu S. Guo Y. Liu J. Wang L. (2025). Human metapneumovirus in children with acute lower respiratory infections: effects on clinical and disease severity. BMC Pediatr. 25, 709. doi: 10.1186/s12887-025-06101-w 41039255 PMC12492612

[B44] Zhang G. Hu Y. Huang B. Gao H. Zhu M. (2024). The predictive value of PCT, SP-D and 8-iso-PGF2 α for the development of severe pneumonia in children. Afr. Health Sci. 24, 63–68. doi: 10.4314/ahs.v24i3.9 40777934 PMC12327119

[B45] Zhang L. Liu W. Liu D. Chen D. Tan W. Qiu S. . (2018). Epidemiological and clinical features of human metapneumovirus in hospitalised paediatric patients with acute respiratory illness: a cross-sectional study in Southern China, from 2013 to 2016. BMJ Open 8, e019308. doi: 10.1136/bmjopen-2017-019308 29437754 PMC5829904

[B46] Zhou H. Haber M. Ray S. Farley M. M. Panozzo C. A. Klugman K. P. (2012). Invasive pneumococcal pneumonia and respiratory virus co-infections. Emerg. Infect. Dis. 18, 294–297. doi: 10.3201/eid1802.102025 22305270 PMC3310442

