# Supplementary material for: Clinical characteristics, co-detection patterns and prognostic risk factors of human metapneumovirus infection in children: a retrospective study
Source: Front Cell Infect Microbiol. 2026 Jul 7;16:1868123. doi: 10.3389/fcimb.2026.1868123 (PMC13384883; doi:10.3389/fcimb.2026.1868123)
Supplement: Supplementary file 1 [file Table1.docx]

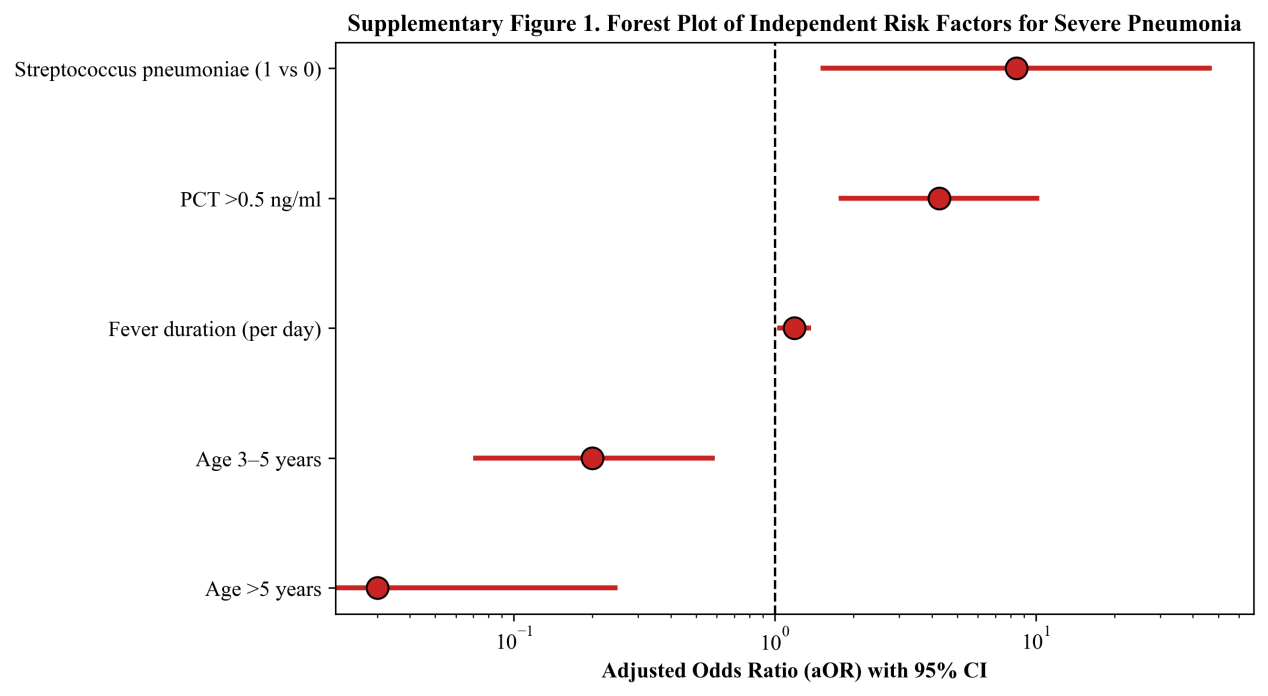
**Supplementary Figure 1**. Forest plot of independent risk factors for severe pneumonia in children infected with human metapneumovirus (hMPV).Adjusted odds ratios (aOR) and corresponding 95% confidence intervals (CI) were calculated using multivariate logistic regression analysis. The vertical dashed line at 1.0 indicates no effect. Variables with aOR > 1 suggest risk factors for severe pneumonia, whereas aOR < 1 indicate protective factors.


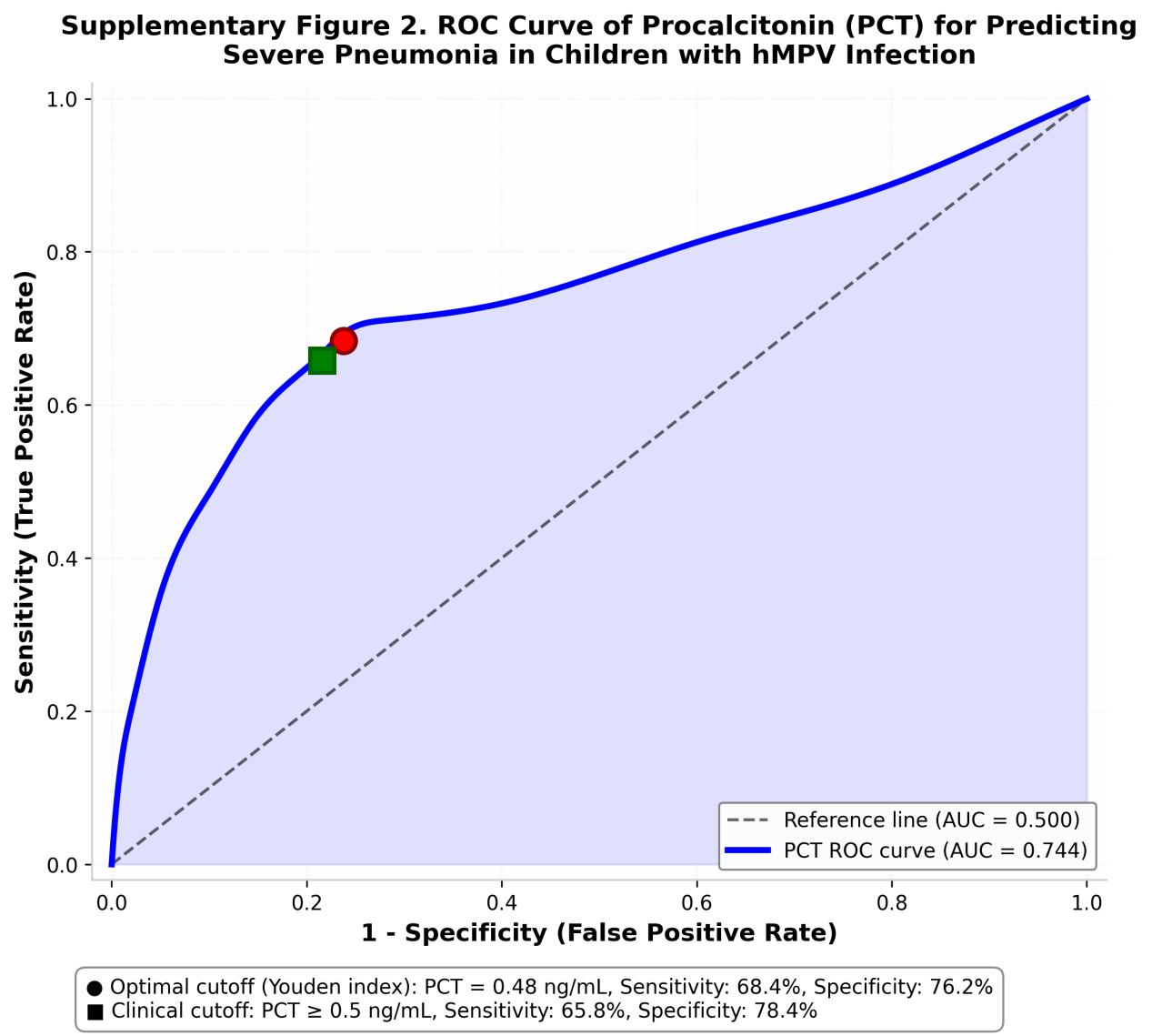


**Supplementary Figure 2**. Receiver operating characteristic (ROC) curve of procalcitonin (PCT) for predicting severe pneumonia in children with human metapneumovirus (hMPV) infection.
